# Subglottic Hemangioma: A Hidden Cause of an Infant’s Respiratory Distress

**DOI:** 10.7759/cureus.65471

**Published:** 2024-07-26

**Authors:** Albaraa Alshalkhaty, Mazen Alhaj Ahmad, Omendra Narayan

**Affiliations:** 1 College of Medicine, University of Sharjah, Sharjah, ARE; 2 Department of Pediatric Pulmonology, American Hospital Dubai, Dubai, ARE

**Keywords:** case report, propranolol, airway obstruction, infant respiratory distress, subglottic hemangioma

## Abstract

Subglottic hemangiomas are uncommon forms of infantile vascular tumors often misdiagnosed due to symptom overlap with other conditions like laryngomalacia, bronchiolitis, and asthma. Early and accurate diagnosis is vital for effective management. This case report discusses a unique presentation of subglottic hemangioma in a three-month-old infant, highlighting its diagnostic challenge and management. It adds valuable insights into the differentiation of subglottic hemangioma from other common causes of respiratory distress in infants.

The infant presented with severe respiratory distress since birth, worsening over the last four weeks, accompanied by gastroesophageal reflux and poor weight gain. Initially, the case was suspected and treated as croup and laryngomalacia. A CT angiogram revealed a vascular lesion in the subglottic area, confirmed by flexible bronchoscopy as a hemangioma. Treatment with propranolol led to significant improvement.

Early diagnosis and treatment of subglottic hemangioma are crucial for a good prognosis. This case emphasizes the importance of considering subglottic hemangioma in infants with unresolved airway distress.

## Introduction

Subglottic hemangioma is a rare infantile condition occurring at a rate of 1.76 cases per 100,000 live births [1]. It is typically asymptomatic, especially during the neonatal period, but can present with biphasic stridor, feeding difficulties, and respiratory distress [2]. This condition is often not diagnosed on the initial presentation, especially when lacking cutaneous signs, as it presents a diagnostic challenge due to overlapping symptoms with more common conditions like croup and laryngomalacia [2,3]. It can be potentially life-threatening especially if complications such as subglottic obstruction arise [2]. The prevalence of obstruction as a complication among infantile hemangioma is 1.5% [4]. Accurate diagnosis is critical for timely treatment, improved outcomes, and avoiding complications. This case report contributes to the medical literature by detailing the diagnostic process and management of subglottic hemangioma, stressing the importance of a multidisciplinary approach.

## Case presentation

A three-month-old infant, born full term with unremarkable nursery stay and no prenatal concerns, had a history of intermittent respiratory distress that was progressively worsening over the past four weeks. She presented with severe respiratory distress that was associated with excessive spit-ups, irritability, and poor weight gain.

The patient presented previously with similar complaints and was suspected to have gastroesophageal reflux, croup, and laryngomalacia on different occasions for which she was prescribed prednisolone and esomeprazole. With her worsening respiratory state, she was referred to a tertiary respiratory pediatric service. Vital signs showed tachypnea and tachycardia, but normal oxygen saturation, temperature, and blood pressure. Clinical assessment showed severe intercostal retractions and inspiratory stridor that aggravated with agitation and alleviated with prone positioning.

**Figure 1:**
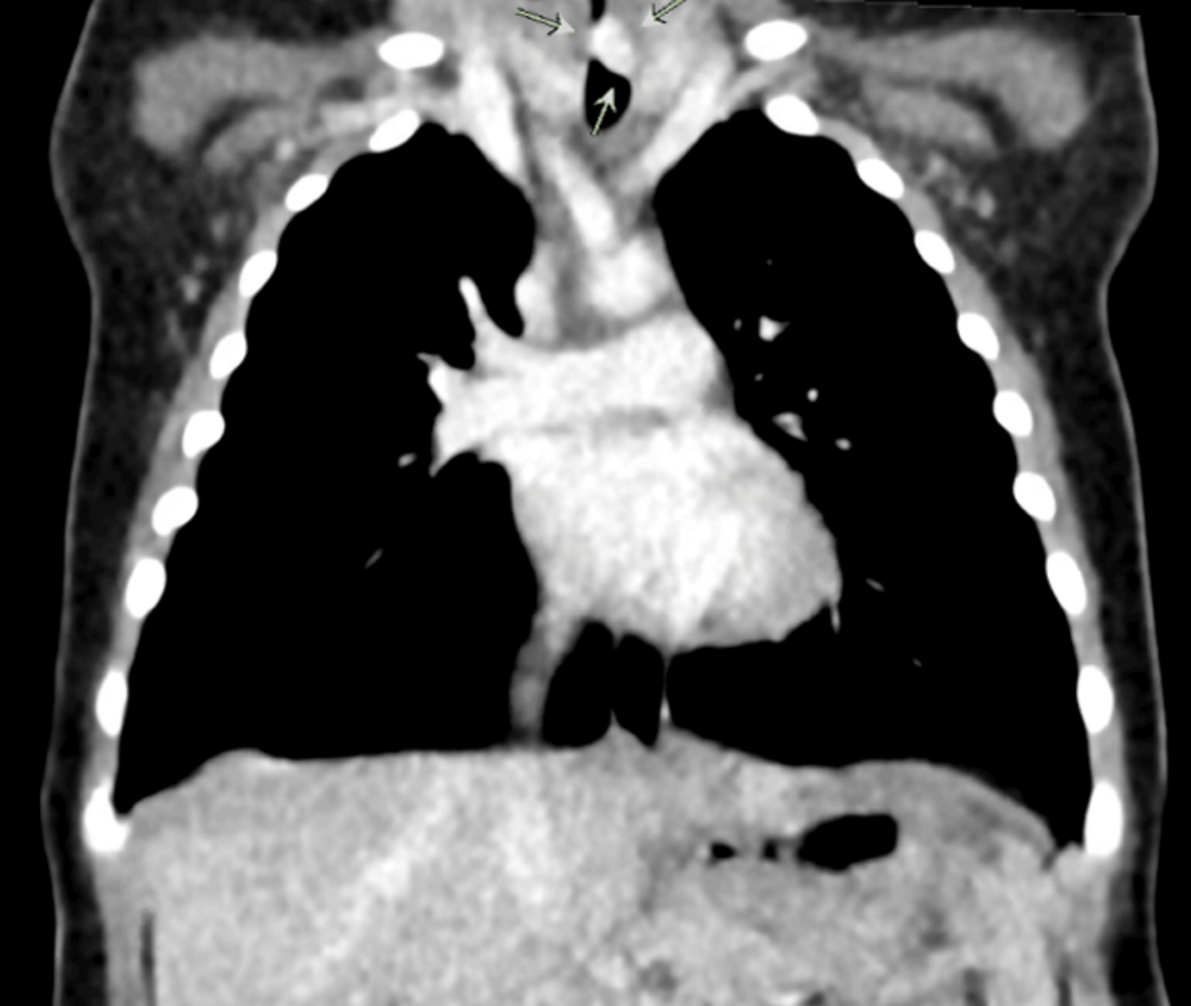
Coronal CT angiogram showing a soft-tissue mass compressing the proximal airway (shown by arrows). CT: computed tomography.

Further investigations were arranged including a CT angiogram that revealed a possible vascular lesion around the subglottic area causing significant compression of the proximal airway (Figure 1).

**Figure 2:**
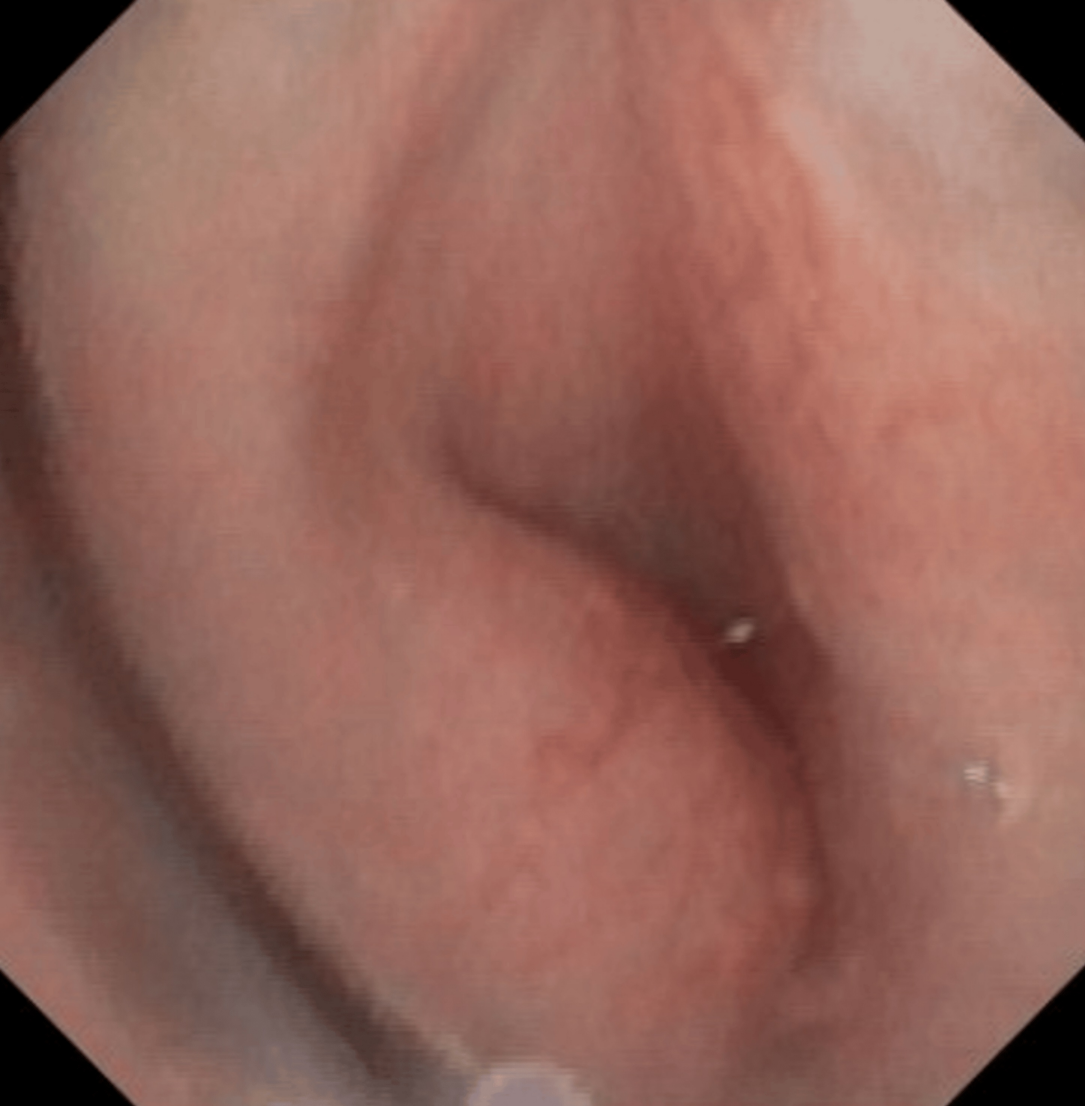
Flexible bronchoscopy of the trachea showing a left-sided hemangioma causing significant obstruction.

Flexible bronchoscopy was later performed confirming that the vascular lesion is a hemangioma almost completely obstructing the trachea (Figure 2). No other hemangiomas were present on examination or imaging modalities.

She was started on propranolol in addition to the continuation of both esomeprazole and prednisolone for the next few days. The propranolol dosage was adjusted based on clinical response, leading to a significant improvement in symptoms. Within the next few days, she had a remarkable improvement on physical examination, and propranolol was gradually weaned in six months. Regular follow-up assessments and imaging confirmed the reduction in hemangioma size. No adverse or unanticipated events were reported.

## Discussion

This case highlights a diagnostic challenge in distinguishing subglottic hemangioma from laryngomalacia due to overlapping symptoms. Early diagnosis of subglottic hemangioma ensures a good prognosis, while delays in either diagnosis or treatment increase mortality rates [5]. A multidisciplinary approach is necessary to achieve a timely diagnosis and treatment [6].

Subglottic hemangiomas have been previously misdiagnosed as bronchitis, pneumonia, laryngomalacia, laryngeal obstruction, and asthma [7]. Therefore, clinicians managing patients with unresolved airway distress should maintain a high index of suspicion for less common causes of stridor. The presentation and management of subglottic hemangioma in this case are consistent with existing literature [1,5], which notes that these hemangiomas are often misdiagnosed due to their rarity and the nonspecific nature of their symptoms. Studies, such as those by Lin et al. (2021) [5] and Chen et al. (2022) [7], emphasize the importance of considering subglottic hemangiomas in infants with persistent respiratory issues. These studies also support the use of propranolol as an effective treatment, which was confirmed in our case with the significant improvement observed in the patient [8,9].

One of the main strengths in the management of this case was the timely referral to a tertiary healthcare center, which facilitated accurate diagnosis and appropriate treatment. The use of advanced imaging techniques such as CT angiogram and flexible bronchoscopy was pivotal in identifying the subglottic hemangioma [6,10]. However, the initial diagnosis of croup and laryngomalacia highlights a significant limitation, as this led to a delay in the correct diagnosis and subsequent treatment [2]. Early consideration of less common causes of respiratory distress could have expedited the proper intervention.

## Conclusions

This case report highlights the clinical presentation, diagnostic process, and management of a less common cause of respiratory distress. Subglottic hemangiomas can also present without cutaneous manifestations and can be confused with other causes such as laryngomalacia and croup. 

The main lessons from this case report include the importance of maintaining a high index of suspicion for less common causes of infant respiratory distress especially when symptoms persist despite standard treatments. Multidisciplinary collaboration and the use of advanced diagnostic tools are essential for accurate diagnosis and effective management. Early diagnosis and intervention can significantly improve outcomes, reducing morbidity and potential mortality associated with delayed treatment.
